# A Novel Method for Carbon Nanotube Functionalization Using Immobilized *Candida antarctica* Lipase

**DOI:** 10.3390/nano12091465

**Published:** 2022-04-26

**Authors:** José Jesús Guzmán-Mendoza, David Chávez-Flores, Silvia Lorena Montes-Fonseca, Carmen González-Horta, Erasmo Orrantia-Borunda, Blanca Sánchez-Ramírez

**Affiliations:** 1 Facultad de Ciencias Químicas, Universidad Autónoma de Chihuahua, Circuito Universitario s/n Campus II, Chihuahua 31125, Mexico; jose.guzmanm@cinvestav.mx (J.J.G.-M.); dchavezf@uach.mx (D.C.-F.); carmengonzalez@uach.mx (C.G.-H.); 2 Tecnologico de Monterrey, Escuela de Medicina y Ciencias de la Salud, Heroico Colegio Militar 4700, Col. Nombre de Dios, Chihuahua 31300, Mexico; silvialorena.montes@tec.mx; 3 Centro de Investigación en Materiales Avanzados (CIMAV), Miguel de Cervantes 120, Complejo Industrial Chihuahua, Chihuahua 31136, Mexico; erasmo.orrantia@cimav.edu.mx

**Keywords:** carbon nanotubes, enzymatic functionalization, *Candida antarctica* lipase, ester bond, covalent functionalization

## Abstract

Carbon nanotubes (CNTs) have been proposed as nanovehicles for drug or antigen delivery since they can be functionalized with different biomolecules. For this purpose, different types of molecules have been chemically bonded to CNTs; however, this method has low efficiency and generates solvent waste. *Candida antarctica* lipase is an enzyme that, in an organic solvent, can bind a carboxylic to a hydroxyl group by esterase activity. The objective of this work was to functionalize purified CNTs with insulin as a protein model using an immobilized lipase of *Candida antarctica* to develop a sustainable functionalization method with high protein attachment. The functionalized CNTs were characterized by scanning electron microscope (SEM), Raman spectroscopy, Fourier-transform infrared spectroscopy (FTIR), thermogravimetric analysis (TGA), and sodium dodecyl sulfate-polyacrylamide gel electrophoresis (SDS–PAGE). The enzymatic functionalization of insulin on the surface of the CNTs was found to have an efficiency of 21%, which is higher in conversion and greener than previously reported by the diimide-activated amidation method. These results suggest that enzymatic esterification is a convenient and efficient method for CNT functionalization with proteins. Moreover, this functionalization method can be used to enhance the cellular-specific release of proteins by lysosomal esterases.

## 1. Introduction

Since their discovery [1], carbon nanotubes (CNTs) have shown a high potential in biological and therapeutic applications [2,3,4,5,6] because they can be functionalized with different molecules by different methods [7,8]; among these, functionalization with proteins has increased their relevance and applications in biological systems. CNTs have been successfully functionalized with proteins, such as ferritin and streptavidin, using a noncovalent method [9]. Additionally, nonspecific adsorption has been used for functionalization with different proteins, such as streptavidin, cytochrome c, bovine serum albumin (BSA), and protein A, among others, attached by noncovalent bonds onto the CNT surfaces. Functionalized CNTs produced by this method can induce biological responses after their internalization, inducing apoptosis by releasing cytochrome c [10]. On the other hand, different studies have focused on the covalent functionalization of CNTs with proteins using a diimide-activated amidation method, attaching amine groups of the proteins to the carboxylic groups present on the CNTs generated by the acid-purification treatment, which is a process that requires the use of toxic cross-linker reagent (N-(3-dimethylaminopropyl)-N-ethyl carbodiimide hydrochloride [11,12]. Covalent immobilization, out of all of these approaches, provides the strongest binding strength between the support nanoparticle and the protein while also reducing leakage and boosting operational stability against heat, pH, organic solvents, and storage [13]. In 2012, acid-treated CNTs were functionalized with an *Entamoeba histolytica* protein (L220) using the amidation method; the authors reported a binding protein yield of 5.28% [14]. Wu et al. (2009) attached the anticancer molecule (10-hydroxycamptothecin; HCPT) to CNTs through amidation with a previously ester-derivatized HCPT, reporting an attaching yield of 16%. Additionally, HCPT-CNTs showed low toxicity, which was attributed to the increase in HCPT release via ester cleavage by cell esterases [15]. Furthermore, antigenic peptides covalently bonded on CNTs are appropriately provided for antibody-specific binding, and CNTs increase immunogenic reactivity without compromising specificity [16]. Consequently, to improve the biological interaction between protein-functionalized CNT cells, it is necessary to increase the quantity of protein attached to CNTs and form a bond susceptible to cleavage by cell enzymes.

*Candida antarctica* lipase (E.C. 3.1.1.3) is an enzyme of the triacylglycerol ester hydrolase family; this enzyme catalyzes the cleavage of carboxylic ester bonds into tri-, di- and monoacylglycerols, releasing the corresponding carboxylic acid in cells. Additionally, under anhydrous conditions or in the presence of traces of water, lipases can catalyze the reverse reaction (esterification), which occurs between a carboxyl group and a hydroxyl group [15,16]. 

Therefore, we propose the use of an immobilized *Candida antarctica* lipase to covalently functionalize multi-walled carbon nanotubes (CNTs) with insulin via ester linkage and to determine the amount of functionalized protein to the CNTs by quantifying the attached protein. Insulin was considered since threonine localized at the C-terminal tail can be susceptible to binding through the hydroxyl group for lipase to the carboxyl groups present on the surface of acid-purified CNTs. Additionally, in theory, proteins attached to the CNT surface through an ester bond can be released by cellular esterases [17,18].

## 2. Materials and Methods

### 2.1. Synthesis and Purification of CNTs

CNTs were synthesized by spray pyrolysis using toluene and ferrocene as the carbon source and catalyst, respectively, following a previously described methodology [19]. In brief, purification was carried out with 0.2 g of CNTs suspended in a mixture of concentrated H_2_SO_4_/HNO_3_ 3:1 *v/v* and sonication. The resultant purified CNTs (CNTs-COOH) were collected by filtration through a 450 nm pore size polytetrafluoroethylene (PTFE) filter, washed four times with water, and methanol, and finally dried at room temperature [20,21]. The unpurified-CNTs and CNTs-COOH were previously characterized as described in previous work [21].

### 2.2. Functionalization of CNTs-COOH with Insulin

**Figure 1:**
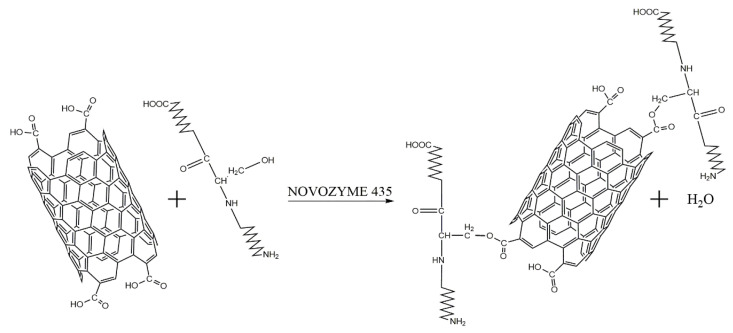
Schematic representation of the functionalization of CNTs using NOVOZYME 435. Acid-treated CNTs display carboxyl groups due to oxidation, and these carboxyl groups are susceptible to reacting with the hydroxyl groups of some amino acids (threonine, serine, or tyrosine) present in proteins—a reaction that is catalyzed by Novozyme 345 (lipase) under anhydrous conditions.

CNT functionalization was realized using an immobilized acrylic resin lipase of *Candida antarctica* (Novozyme 435, Sigma–Aldrich, St. Louis, MO, USA). Lipase can catalyze esterification between the carboxylic group present in the CNTs-COOH and a hydroxyl group present in some amino acids (Figure 1).

For this purpose, 10 mg of CNTs-COOH was suspended in a vial with 10 mL of cyclohexane (Sigma–Aldrich), 130 µg of commercial human insulin (PHARMAlife^®^, Zapopán, JAL, Mexico) and 20 mg of Novozyme 435 were added and stirred; the reaction was carried out at 60 °C with constant stirring for 24 h. After incubation, the mix reaction presented the following two phases: CNTs on the superior layer were recovered by filtration through a 0.2 μm pore size PTFE filter and identified as CNTs-INSs. The CNTs on the bottom phase were resuspended in absolute ethanol and identified as CNTs-INSp. Both types of CNTs-INS were washed three times with ethanol to remove the cyclohexane and centrifuged at 4500× *g* for 30 min at 4 °C, followed by three washes with water to remove the ethanol; the resulting CNT-INS were lyophilized. The immobilized enzyme was removed with dissection tweezers and reutilized in other reactions.

Cyclohexane was used because this nonpolar solvent generates an anhydrous condition that is optimum for the esterification reaction, and it has no functional groups that can interfere with the reaction [22,23].

As reaction controls, we performed the same reaction, with all the described elements but (1) without enzyme, as covalent functionalization control was carried out by the lipase (CNTs-WO/Enzy), and (2) without protein, as an auto functionalization control (CNTs-WO/Prot).

The functionalization efficiency was calculated (Equation (1)) by quantifying the total protein in the functionalized sample after all washes using the Bradford and bicinchoninic acid (BCA) method [24,25]. The protein concentration was determined in pristine CNTs, CNT-COOH, CNT-WOEnzy, and WO/Prot. As a blank, the absorbance of CNT-COOH at the same concentration was measured and subtracted from all CNT-INS samples and controls.

(1) $$\mathrm{Functionalization}\;\mathrm{efficiency}=\frac{\mathrm{Quantified}\;\mathrm{protein}\;\mathrm{in}\;\mathrm{the}\;\mathrm{functionalized}\;\mathrm{CNTs}\;\left(µg\right)}{\mathrm{Total}\;\mathrm{protein}\;\mathrm{added}\;\mathrm{to}\;\mathrm{the}\;\mathrm{reaction}\;\left(µg\right)}\times 100$$

### 2.3. Characterization of CNTs

The different CNTs were characterized by scanning electron microscopy using a JEOL SEM, model JSM-5800 LV.

Raman spectroscopy was performed using a micro-Raman LabRAM HR (Horiba Jobn Yvon, Piscataway, NJ, USA) coupled to an Olympus BX-4 microscope (Olympus, Miami, FL, USA) and Spectrum Gx (Perkin Elmer, Waltham, MA, USA). The sample was excited using a laser line at a wavelength of 632.8 nm, and all measurements were performed at room temperature.

The functional groups present in different CNTs were determined by Fourier Transform Infrared (FTIR) spectroscopy in transmittance mode using a Carry 600 Series FTIR Spectrometer (Agilent Technologies, Santa Clara, CA, USA) equipped with a zinc selenide accessory in attenuated total reflectance (ATR) mode; the wavenumber ranged of 600 cm^−1^ to 4000 cm^−1^. CNTs were deposited as dry powders onto a zinc selenide (ZnSe) window, no solvents were used in this process, and all determinations were made at room temperature.

A thermogravimetric analysis (TGA) was performed using a SDT Q600 V20.9 Build 20 (TA Instruments, New Castle, DE, USA) heated at a rate of 10 °C/min to 1000 °C, and air was introduced into the samples at a rate of 25 mL/min.

Finally, 10% SDS–PAGE was carried out, and the proteins were visualized using the ProteoSilver™ Silver Stain Kit (Sigma–Aldrich) to demonstrate the presence of proteins in the sample.

## 3. Results and Discussion

### 3.1. Carbon Nanotube Synthesis

**Figure 2:**
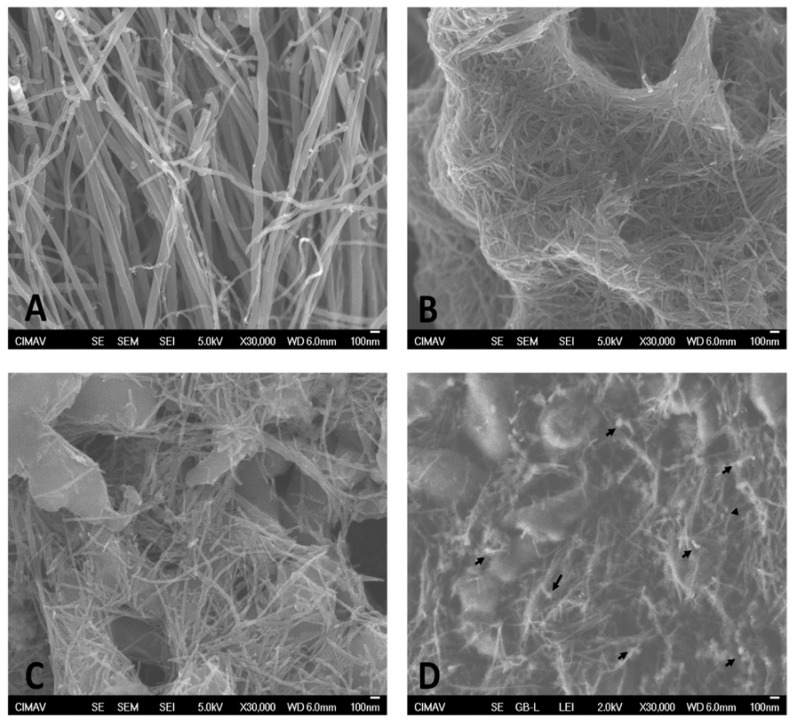
Microphotographs obtained by SEM. Microphotographs show the aspects of (**A**) pristine CNTs, (**B**) purified CNT-COOH, (**C**) CNT-INSp, and (**D**) CNT-INSs. Arrows indicate spherical bodies detected on the CNT surface.

After synthesis, pristine CNTs (CNTs) (Figure 2A) had a length and diameter of ≈30 µm and ≈70 nm, which was decreased to ≈500 nm and ≈26 nm, respectively, after the acid treatment (Figure 2B), in accordance with our previous report [21].

### 3.2. SEM Analysis of Functionalized Carbon Nanotubes

Figure 2 depicts an SEM analysis of the different CNTs. Functionalized CNTs were separated according to their solubility in CNTs-INSp (Figure 2C) and CNTs-INSs (Figure 2D) obtained in the precipitated cyclohexane and the supernatant, respectively.

As shown in Figure 2D, we observed some spherical aggregates with a low electron density on the surface of CNT-INSs, which is attributed to the attached insulin [26]. Moreover, CNTs-INSp presented fewer spherical aggregates, which suggests lower functionalization in these CNTs (Figure 2C).

Raman spectroscopy, FTIR, and TGA were performed to confirm the functionalization.

### 3.3. Raman Analysis of Functionalized Carbon Nanotubes

Raman spectroscopy was used to characterize the surface modification of CNTs. The characteristic Raman spectra for CNTs showed the following three bands: the G band at 1600 cm^−1^, which corresponds to the fundamental vibration of the tangential elongation, and another band at 2650 cm^−1^, which corresponds to a second-order overtone (G′ band). In addition, CNTs show a band at 1300 cm^−1^, called the band D band (“Induced disorder”), which is indicative of defects at the surfaces of the walls and is an indicator of the presence of structural defects [27].

**Figure 3:**
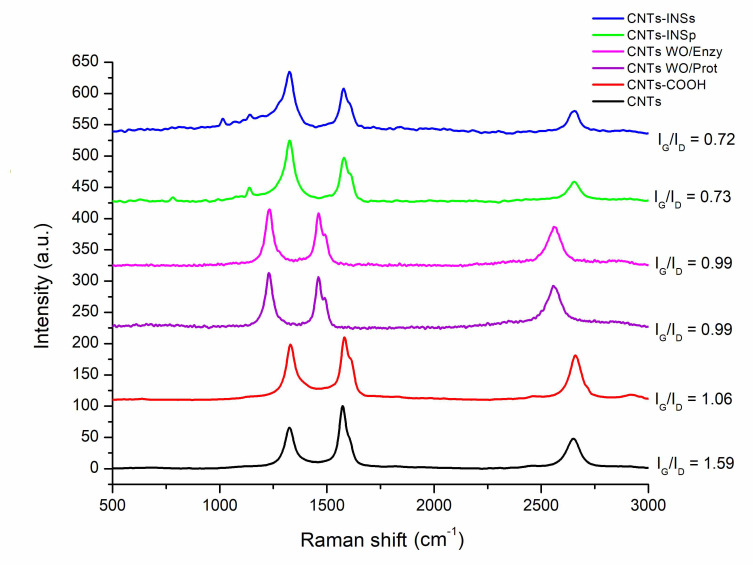
Raman spectra for the different CNTs. In each figure, the D band is shown at 1338 cm^−1^, and the G band is shown at 1600 cm^−1^ (laser excitation at 632.8 nm).

Figure 3 displays the Raman spectra for each CNT, and the ratio between the intensities of the G and D bands (I_G_/I_D_) was calculated and included in the figure. The I_G_/I_D_ ratio is related to the structural quality; pristine CNTs show the highest I_G_/I_D_ ratio, followed by CNTs-COOH. Functionalization with insulin decreases the structural quality of the CNTs, reducing the I_G_/I_D_ ratio to 0.72 since the added insulin disturbs the surface of the CNTs, and interactions of radical insulin groups with carbonyl or carboxyl groups on CNTs-COOH can also be implicated. Additionally, in the CNTs-INSs, a clear band at 1000 cm^−1^ was observed, which corresponds to the insulin spectra [28].

Figure 3 depicts the reaction controls (CNTs-WO/Enzy and WO/Prot) and their I_G_/I_D_ ratios, which do not show significant differences compared with CNTs-COOH.

### 3.4. FTIR Analysis of Functionalized CNTs

Fourier transform infrared spectroscopy (FTIR) was performed to determine the functional groups present on the surface of the CNTs.

**Figure 4:**
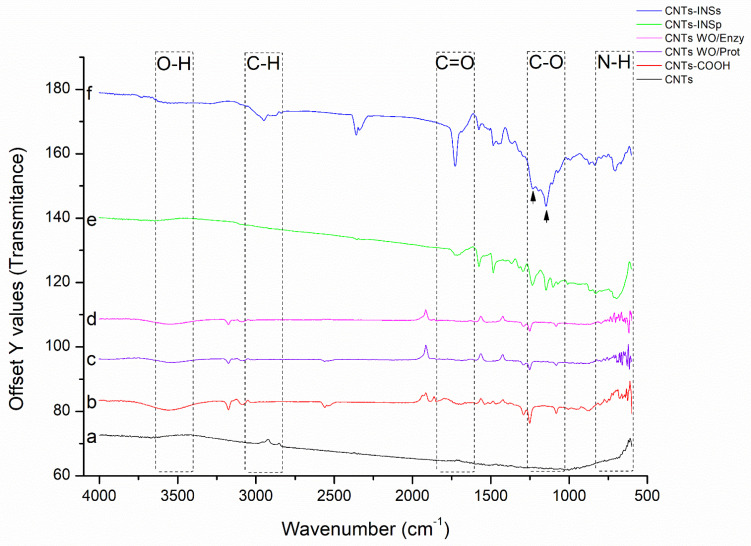
FTIR for the different CNTs. The signals due to the C=O bond (1733 cm^−1^), C-O (1146 and 1239 cm^−1^), O-H (3300–3600 cm^−1^), N-H (700 cm^−1^) as well as C-H stretching (2875–2950 cm^−1^) are shown.

The FTIR spectra for the different CNTs are shown in Figure 4. Pristine CNTs show a characteristic baseline without peaks since the CNTs initially do not present any functional groups on their surface (Figure 4 (a), black line). After purification with acid treatment, peaks corresponding to the COOH group (C=O and O-H) and the C-C bond of the carboxyl group attached to the CNTs (Figure 4 (b), red line) were detected (also, see Supplementary Figure S1). On the other hand, for the CNT-INSs (Figure 4 (f), blue line), an intense peak corresponding to the C=O group was observed at 1733 cm^−1^, as well as two other signals corresponding to two C-O bonds, indicating an ester bond (COO-R; arrows); besides, the broadband between 3000 and 3500 cm^−1^ decreases significantly suggesting a reduction in free carboxylic groups and corroborate the bond between insulin and CNTs-COOH. Likewise, signals corresponding to CH3 and CH2 are observed (a region that is characteristic of amino acids) [29] in the CNTs-INSs, corresponding to the amino acids of insulin; these signals are absent in the CNTs-COOH and CNTs-INSp (Figure 4 (b,e), red and green line, respectively). The controls (Figure 4 (c,d), lines purple and pink, respectively) show a similar spectrum to CNTs-COOH (Figure 4 (b), red line).

### 3.5. Thermogravimetric Analysis of Functionalized CNTs

**Figure 5:**
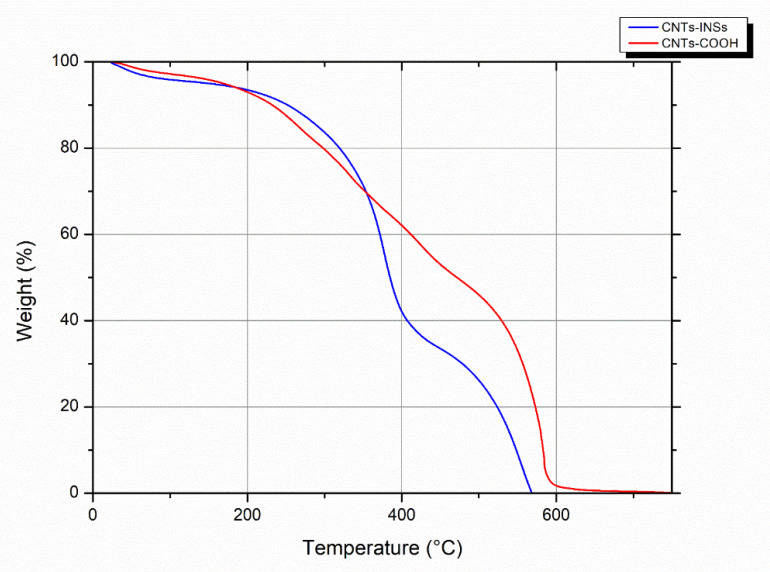
Thermogravimetric analysis of functionalized CNTs.

A TGA analysis was performed to confirm and quantify the level of functionalization through the weight loss of the bound protein on the CNTs-COOH. The results are displayed in Figure 5; a weight loss of approximately 20% at 400 °C for CNT-INSs (blue line) was detected compared with CNTs-COOH (red line).

On the other hand, TGA of CNTs has become a relevant tool for purity analysis. The residual mass data provide information for the decomposition of the material. Most carbon-based materials are decomposed at 650 °C, and the residual mass is attributed to the metal catalyst particles that remain [30]. As shown in Figure 5, at 650 °C, the CNTs-COOH and CNT-INSs have no residual mass at this temperature, indicating very low or nonmetal catalysis particles resulting from a good purification process.

### 3.6. Protein Quantification and SDS–PAGE of Functionalized CNTs

Finally, the functionalization efficiency was calculated using the Bradford and BCA method in each of the different functionalized CNTs by calculating the total protein in each of the functionalized samples after all stages of washing. The results showed undetected amounts of protein in CNTs-WO/Prot, CNTs-WO/Enzy (indicating nonauto functionalization and nonprotein adsorption on CNTs). Protein traces were found in CNTs-INSp (0.0004 mg of protein by BCA method) and the functionalization efficiency was 0.34%, suggesting that these CNTs are poorly functionalized, agreeing with the FTIR spectrum and SDS-PAGE.

Regarding the CNT-INSs, the functionalization efficiency calculated based on the amount of protein in the sample was 21%. Moreover, this efficiency can be improved since, in this study, we used only one concentration of insulin in the reaction. These results are in accordance with the TGA analysis (Figure 5), which shows a weight loss for CNT-INSs of approximately 20% compared with the nonfunctionalized sample.

**Figure 6:**
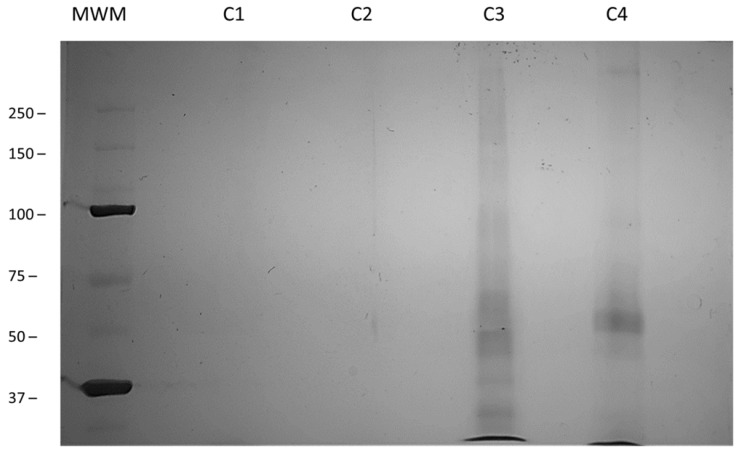
SDS–PAGE of functionalized CNTs. MWM; molecular weight marker, C1; CNTs without enzyme, C2; CNTs without protein, C3; CNTs-INSs, C4; CNTs-INSp. 10% SDS–PAGE. Silver staining.

In addition, protein attachment to the CNTs was confirmed by 10% SDS–PAGE and silver staining, as reported previously [29]. The separation of CNT-INSs based on their charge and length is displayed in Figure 6. CNTs-WO/Enzy or protein (Figure 6, C1 and C2) did not show proteins in the sample in accordance with all previous results. Figure 6, C3, and C4 (CNT-INSs and INSp, respectively) show a multiband pattern, possibly attributed to the different lengths of functionalized CNTs or different protein aggregates. High molecular weight bands can be generated by longer CNT-INSs with a high amount of insulin, whereas low molecular weight bands can be generated by shorter CNTs with less insulin [29]. Additionally, CNT-INSp shows less protein in SDS-PAGE, which is in accordance with BCA and Bradford results.

## 4. Conclusions

These results demonstrate that functionalization using enzymatic esterification is a suitable and sustainable method that produces higher efficiency than conventional chemical methods. In addition, enzymes are reusable, can be used in several reactions, and reduce solvent waste. Moreover, this functionalization can be used for the specific release of proteins in cells since proteins can be released by cellular esterases. Thorough studies need to be performed to confirm this possibility. Furthermore, additional experiments are necessary to determine whether insulin attached to CNT is active in biological systems.

## Supplementary Materials

The following supporting information can be downloaded at: https://www.mdpi.com/article/10.3390/nano12091465/s1, Figure S1: FTIR spectra of different CNTs.
