# Supplementary material for: A Novel Method for Carbon Nanotube Functionalization Using Immobilized Candida antarctica Lipase
Source: Nanomaterials (Basel). 2022 Apr 26;12(9):1465. doi: 10.3390/nano12091465 (PMC9105613; doi:10.3390/nano12091465)
Supplement: Supplementary file 1 [file nanomaterials-12-01465-s001.zip › nanomaterials-1617175-supplementary.pdf]

Supplementary Materials

A Novel Method for Carbon Nanotube Functionalization Using Immobilized *Candida antarctica* Lipase

José Jesús Guzmán-Mendoza [1], David Chávez-Flores [1], Silvia Lorena Montes-Fonseca [2], Carmen González-Horta [1], Erasmo Orrantia-Borunda [3] and Blanca Sánchez-Ramírez [1,*]

1 Facultad de Ciencias Químicas, Universidad Autónoma de Chihuahua, Circuito Universitario s/n Campus II, Chihuahua 31125, Mexico; jose.guzmanm@cinvestav.mx (J.G.-M.); dchavezf@uach.mx (D.C.-F.); carmengonzalez@uach.mx (C.G.-H.)

2 Tecnologico de Monterrey, Escuela de Medicina y Ciencias de la Salud, Heroico Colegio Militar 4700, Col. Nombre de Dios, Chihuahua 31300, Mexico; silvialorena.montes@tec.mx

3 Centro de Investigación en Materiales Avanzados (CIMAV), Miguel de Cervantes 120, Complejo Industrial Chihuahua, Chihuahua 31136, Mexico; erasmo.orrantia@cimav.edu.mx

* Correspondence: bsanche@uach.mx; Tel.: +52-614-614-255-0177

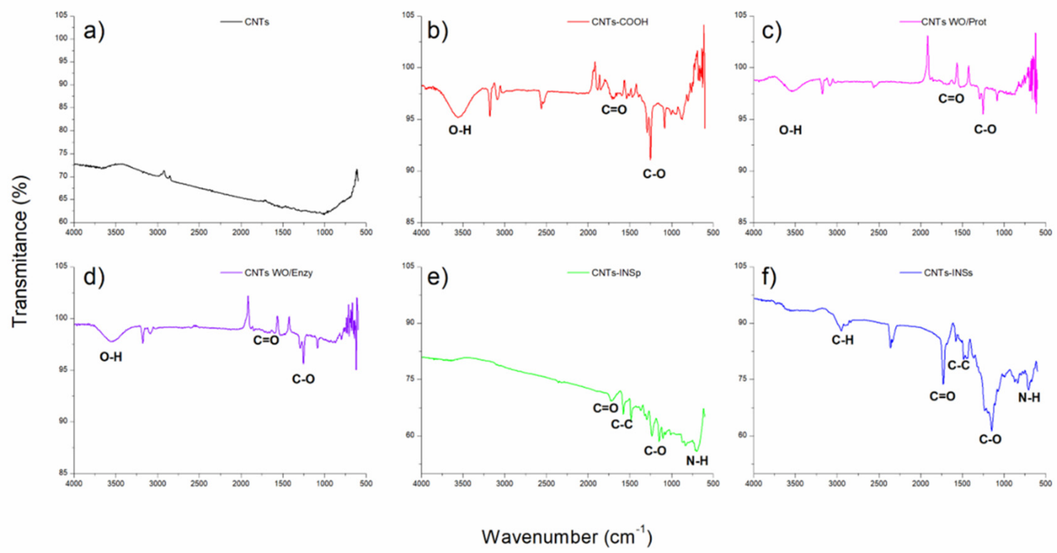

Figure S1. FTIR spectra of different CNTs. (**a**) Pristine CNTs, (**b**) CNTs-COOH, (**c**) CNTs without protein, (**d**) CNTs without enzyme, (**e**) Insulin-functionalized CNTs obtained in the precipitated, (**f**) Insulin-functionalized CNTs obtained in the supernatant. The signals due to the C = O bond (1733 cm-1), C-O (1146 and 1239 cm-1), O-H (3300-3600 cm-1), N-H (700 cm-1) as well as C-H stretching (2875-2950 cm-1) are shown.
